# Cellular microRNA-155 Regulates Virus-Induced Inflammatory Response and Protects against Lethal West Nile Virus Infection

**DOI:** 10.3390/v12010009

**Published:** 2019-12-19

**Authors:** Janhavi P. Natekar, Hussin A. Rothan, Komal Arora, Philip G. Strate, Mukesh Kumar

**Affiliations:** Department of Biology, College of Arts and Sciences, Georgia State University, Atlanta, GA 30303, USA; jnatekar1@student.gsu.edu (J.P.N.); hrothan@gsu.edu (H.A.R.); karora@gsu.edu (K.A.); pstrate1@gsu.edu (P.G.S.)

**Keywords:** West Nile virus, micro-RNAs, miR-155, immune response, inflammatory cytokines and chemokines, virus replication

## Abstract

West Nile virus (WNV) is a flavivirus that has disseminated globally as a significant cause of viral encephalitis in humans. MircoRNA-155 (miR-155) regulates various aspects of innate and adaptive immune responses. We previously reported that WNV infection induces upregulation of miR-155 in mice brains. In the current study, we demonstrate the critical role of miR-155 in restricting the pathogenesis of WNV infection in mice. Compared to wild-type (WT) mice, miR-155 knockout mice exhibited significantly higher morbidity and mortality after infection with either a lethal strain, WNV NY99, or a non-lethal strain, WNV Eg101. Increased mortality in miR-155^−/−^ mice was associated with significantly high WNV burden in the serum and brains. Protein levels of interferon (IFN)-α in the serum and brains were higher in miR-155^−/−^ mice. However, miR-155^−/−^ mice exhibited significantly lower protein levels of anti-viral interleukin (IL)-1β, IL-12, IL-6, IL-15, and GM-CSF despite the high viral load. Primary mouse cells lacking miR-155 were more susceptible to infection with WNV compared to cells derived from WT mice. Besides, overexpression of miR-155 in human neuronal cells modulated anti-viral cytokine response and resulted in significantly lower WNV replication. These data collectively indicate that miR-155 restricts WNV production in mouse and human cells and protects against lethal WNV infection in mice.

## 1. Introduction

West Nile virus (WNV) is a flavivirus that causes severe encephalitis in humans and horses. WNV is maintained in an enzootic cycle between mosquitoes and birds [1,2]. Persistence of WNV infection can cause long-lasting sequelae such as a chronic renal disease [3,4]. The continuing outbreaks of WNV-associated neurological disease underscore the urgent need for effective anti-viral strategies. MicroRNAs (miRNAs) are a group of small RNAs involved in the regulation of several pathways including cell cycle, apoptosis, and immune response [5,6]. miRNAs are considered novel diagnostic and interventional candidate(s) due to their biochemical structure. Multiple studies have reported that miRNAs possess a fundamental role in host-viral interactions as the miRNAs of infected cells can influence the ability of the virus to replicate or spread [7,8,9]. It is known that endogenous miRNAs inhibit replication of a number of RNA viruses including HIV-1, Ebola virus and vesicular stomatitis virus [10,11,12,13]. Over-expression of miRNA-30e, let-7c, and miRNA-126-5p inhibits dengue virus (DENV) replication [14,15,16]. Cellular miR-532-5p inhibits WNV replication via suppression of host genes SESTD1 and TAB3 required for virus replication [17]. Moreover, incorporation of a target sequence for cellular microRNAs expressed in the brain into the flavivirus genome alters the neurovirulence of the virus and prevents development of lethal encephalitis in mice [18]. 

miR-155 is multifunctional and modulates various aspects of innate and adaptive immune responses [12,13,19]. miR-155 plays a crucial role in regulating toll-like receptor-mediated innate immune response and also targets complement regulatory proteins and enhances complement activation [19,20,21]. Several published studied have demonstrated the important role of miR-155 in viral infections. For example, overexpression of miR-155 led to significant reduction in human HIV replication in macrophages [12]. It has been reported that miR-155 regulates viral infections caused by Epstein–Barr [22], Borna disease [10], and reticuloendotheliosis viruses [11,23]. miR-155 suppresses Japanese encephalitis virus (JEV) replication in microglial cells and regulates JEV-induced inflammatory response in mice brains [24,25]. 

We previously reported that WNV infection induces significant upregulation of miR-155 in mice brains [26]. In the current study, we demonstrate the critical role of miR-155 in restricting the pathogenesis of WNV infection in mice. The miR-155 knockout mice exhibited severe neurological disease compared to wild-type (WT) mice after infection with a lethal (WNV NY99) or a non-lethal (WNV Eg101) WNV strain. miR-155 impacts WNV pathogenesis and resistance by regulating anti-viral cytokine and chemokine responses. Bone marrow-derived macrophages (BMDMs) and mouse embryonic fibroblasts (MEFs) lacking miR-155 were more susceptible to infection with WNV compared to cells derived from WT mice. In addition, overexpression of miR-155 in human neuronal cells modulated anti-viral cytokine response and resulted in significantly lower WNV replication. Collectively, these data provide the first evidence of the requirement for miR-155 as a critical host factor for restricting WNV infection. 

## 2. Materials and Methods

### 2.1. Ethics Statement

C57BL/6 (WT) and miR-155 knockout mice (miR-155^−/−^ mice) on C57BL/6 background were obtained from The Jackson Laboratory. The animal experiments were conducted in the animal biosafety level-3 laboratory according to the guidelines of the Institutional Animal Care and Use Committee at Georgia State University (Protocol number A19006, Approval date 09/01/2018) and the National Institutes of Health. 

### 2.2. Animal Infections with WNV

Eight-week-old WT and miR-155^−/−^ mice were subcutaneously injected in footpads with 100 plaque-forming units (PFU) of WNV NY99 or 1000 PFU of WNV Eg101 [27,28,29,30]. Animals were observed twice a day for clinical signs such as ruffled fur, hunchbacked posture, paralysis, tremors and ataxic gait. Animals displaying severe clinical signs were euthanized to limit suffering. At various time points after virus infection, blood was collected from the tail vein and serum was separated. In a separate set of experiments, mice were inoculated with PBS (Mock) or WNV NY99 (100 PFU) or WNV Eg101 (1000 PFU), and mice were sacrificed, and their brains harvested at day 8 after infection. WNV titers in the mice serum and brain homogenates were measured by plaque formation assay [29].

### 2.3. ELISA and Multiplex Immunoassay 

Luminex assay was used to determine the protein levels of cytokines and chemokines in the serum using a Milliplex Map Mouse Cytokine/Chemokine kit (Millipore, Massachusetts, USA) [31]. The levels of alpha interferon (IFN-α) were determined in the mice serum and brain homogenates by VeriKine Mouse Interferon-Alpha ELISA Kit (PBL Interferon Source, Piscataway, NJ, USA) [31].

### 2.4. WNV Infection in Primary Mouse Cells

MEFs were isolated from 1-day-old pups [31,32,33,34]. MEFs were grown in DMEM (Thermo Fisher Scientific, Norcross, GA, USA) supplemented with 10% FBS and 10 μg/mL gentamicin (Thermo Fisher Scientific, Norcross, GA, USA). For BMDM isolation, eight-week-old miR-155^−/−^ and WT mice were euthanized, and bone marrow cells were isolated from the hind limbs as described previously [28]. The cultures were maintained in DMEM containing 10% FBS, and 40 ng/mL macrophage colony-stimulating factor (R&D Systems, Minneapolis, MN, USA) for one week before WNV infection. Both BMDMs and MEFs were infected with either WNV NY99 or WNV Eg101 at a multiplicity of infection (MOI) of 1. Cell culture supernatants and cell lysates were collected at various time points after infection. WNV titers were measured in the culture supernatants by plaque formation assay [28,33,35]. 

### 2.5. miRNA Overexpression in Human Neuroblastoma Cell Line, SK-N-SH

SK-N-SH cells were transfected with 100 pmol of miScript miR-155 mimic (Qiagen, Germantown, MD, USA) or miRNA mimic control (Qiagen, Germantown, MD, USA) using Opti-MEM medium (Invitrogen, Carlsbad, CA, USA) and Lipofectamine 2000 (Invitrogen, Carlsbad, CA, USA) [25]. After 24 h of transfection, the cells were infected with WNV NY99 at a MOI of 1. Cell culture supernatants and cell lysates were collected at various time points after infection. Virus titers in culture supernatants were measured by plaque formation assay. 

### 2.6. qRT-PCR

qRT-PCR was conducted on cell lysates from mock and WNV-infected cells to determine the expression of various pro-inflammatory cytokines. Total RNA was extracted from cell lysates using a RNeasy Mini Kit (Qiagen, Germantown, MD, USA). One microgram of RNA was reverse transcribed to cDNA using a iScript^TM^ cDNA Synthesis Kit (Bio-Rad, Des Plaines, IL, USA) [28,36]. The primer sequences are listed in Table 1. 

### 2.7. Statistical Analysis 

Log-rank (Mantel-Cox) Test and Gehan-Breslow-Wilcoxon Test were used to analyze differences in the survival between WT and miR-155^−/−^ mice. Comparison of means was carried out with unpaired Student *t* test. For virus titers in cell culture supernatants, two-way analysis of variance (ANOVA) with the post hoc Bonferroni test was used. *p* values of <0.05 were considered significant.

## 3. Results

### 3.1. MicroRNA-155 Protects against Lethal WNV NY99 Infection

**Figure 1:**
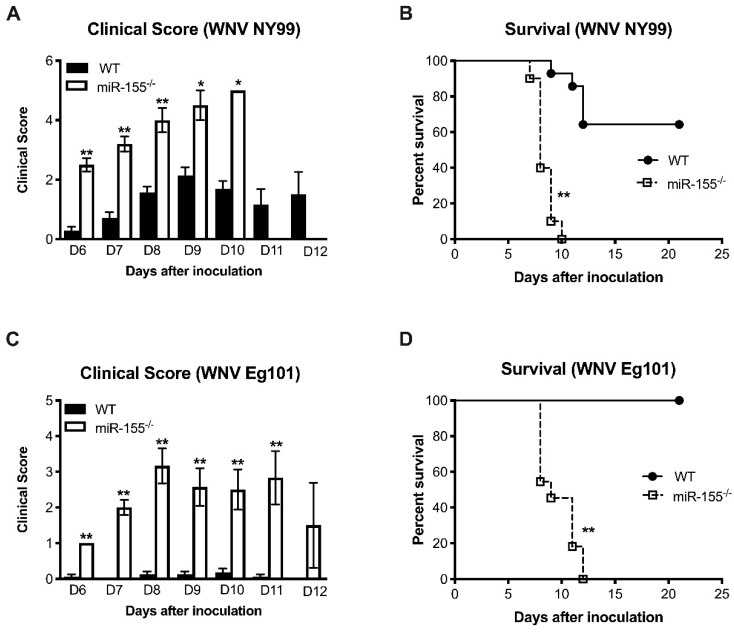
Clinical scores and survival analysis of West Nile virus (WNV) NY99 and WNV Eg101 infected WT and miR-155^−/−^ mice. (**A**,**B**) WT and miR-155^−/−^ mice were monitored twice daily for clinical signs as described in the materials and methods. Error bars represent SEM, * *p* < 0.05, ** *p* < 0.001. (**C**,**D**) The statistical differences in the survival of WT and miR-155^−/−^ mice were significant for both WNV NY99 and WNV Eg101 (*n* = 20 per group for WNV NY99 and *n* = 12 per group for WNV Eg101). ** *p* < 0.001.

We first examined the survival of mice deficient in miR-155 against a sublethal dose of a pathogenic WNV NY99 strain. Wild-type (WT) and miR-155^−/−^ mice were inoculated subcutaneously with 100 PFU of WNV NY99 and monitored for 25 days after inoculation. Mice were monitored for clinical signs that include ruffled fur, hunchbacked posture, paralysis, tremors, and ataxic gait. miR-155^−/−^ mice were highly susceptible to WNV NY99 infection and exhibited significantly higher morbidity than WT mice. As depicted in Figure 1A, mice lacking miR-155 developed severe neurological signs after infection with WNV NY99 compared to the WT mice. All WNV NY99-infected miR-155^−/−^ mice met humane endpoints and were euthanized. Only 35% of WT mice were euthanized during the study period (Figure 1B). The difference in the survival between WT and miR-155^−/−^ mice was statistically significant. 

### 3.2. MicroRNA-155 is Required for Survival after Non-Lethal WNV Eg101 Challenge

To understand the role of miR-155 in restricting lethal WNV encephalitis, we inoculated WT and miR-155^−/−^ mice subcutaneously with 1000 PFU of a non-pathogenic WNV Eg101 strain. WNV Eg101 is largely non-pathogenic in adult mice after subcutaneous inoculation [30]. As expected, no morbidity was observed in WT mice infected with WNV Eg101 (Figure 1C). However, all the miR-155^−/−^ mice developed severe neurological signs after infection with WNV Eg101. All infected miR-155^−/−^ mice were euthanized by day 12 after infection (Figure 1D). These data collectively suggest that miR-155 is critical for the control of WNV infection and pathogenesis in infected mice.

### 3.3. miR-155 Modulates WNV Replication and Neuroinvasion

**Figure 2:**
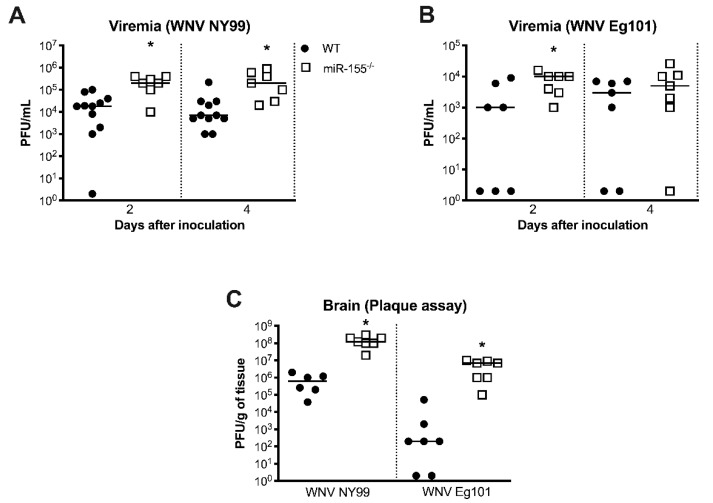
Virus load in the serum and brains of WNV-infected miR-155^−/−^ and WT mice. (**A**,**B**) Virus titers (plaque-forming units (PFU)/mL) were assessed in the serum at days 2 and 4 after WNV NY99 or WNV Eg101 inoculation by plaque assay. (**C**) Virus titers (PFU/g of tissue) were measured in the brains at day 8 after inoculation with WNV NY99 or WNV Eg101. Each data point represents an individual mouse. The solid horizontal lines signify the median. * *p* < 0.05.

To further understand how the deficiency of miR-155 caused severe disease following WNV infection, we measured the viral loads in serum at various time points after inoculation. Plaque assay data showed significantly higher viremia in miR-155^−/−^ mice than WT mice at days 2 and 4 after infection with WNV NY99 (Figure 2A). Similarly, virus titers were significantly higher in miR-155^−/−^ mice at day 2 after infection with WNV Eg101. However, there was no statistically significant difference in virus titers between both the groups at day 4 after WNV Eg101 infection (Figure 2B). We next determined virus titers by plaque assay in the brains harvested at day 8 after infection. It is known that peak virus load is observed at day 8 after WNV infection in the mice [30]. Virus titers in the brains of miR-155^−/−^ mice were significantly higher after infection with WNV NY99 or WNV Eg101 (Figure 2C). Thus, the absence of miR-155 caused increased virus replication and neuroinvasion after WNV infection. 

### 3.4. Antiviral Interferon Response is Altered in miR-155^−/−^ Mice

**Figure 3:**
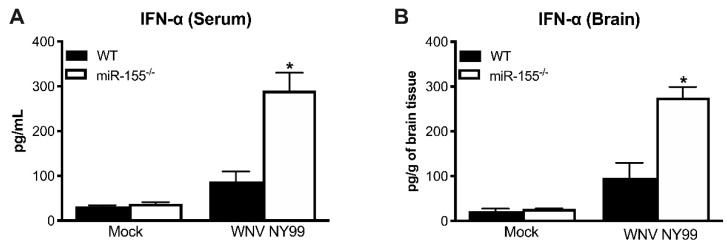
Levels of interferon (IFN)-α in WT and miR-155^−/−^ mice following WNV NY99 infection. (**A**) Protein levels of IFN-α were assessed in the mice serum at day 3 after inoculation and expressed as pg/mL of serum. (**B**) IFN-α levels were measured in brain homogenates at day 8 after inoculation and expressed as pg/g of brain tissue. Error bars represent SEM (*n* = 6–8 mice per group). * *p* < 0.05.

The IFN response is crucial for the host defense against WNV infection [37]. Recent reports have demonstrated that miR-155 plays a significant role in regulating type I IFN response [21,38]. Thus, we next examined the protein levels of IFN-α in the periphery and CNS during WNV NY99 infection in the presence or absence of miR-155. Interestingly, miR-155^−/−^ mice exhibited significantly higher levels of IFN-α in the serum compared to WT mice after WNV NY99 infection (Figure 3A). Similarly, significantly higher IFN-α levels were detected in the brains of miR-155^−/−^ mice infected with WNV NY99 compared to WT mice (Figure 3B). These data indicate that miR-155 may act as a negative regulator of the type I IFN response during WNV infection. It is also possible that high virus replication in miR-155^−/−^ mice resulted in a higher interferon response in these mice. 

### 3.5. miR-155 Regulates WNV-Induced Inflammatory Response 

**Figure 4:**
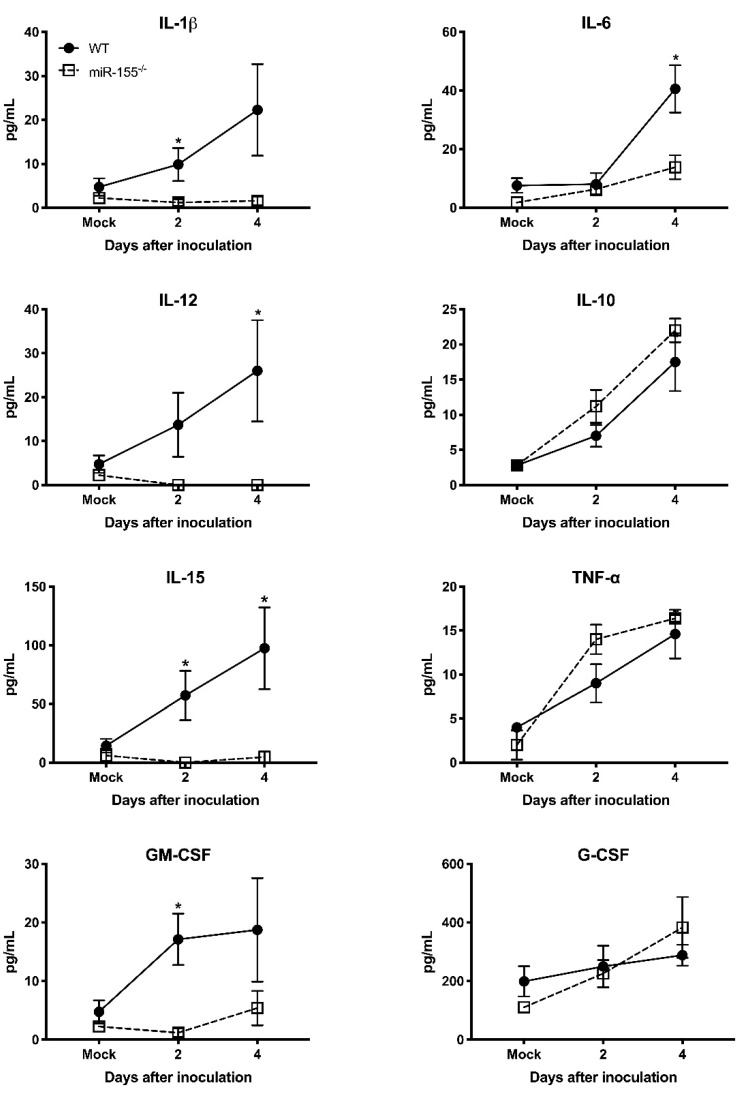
Serum protein levels of cytokines in the miR-155^−/−^ and WT mice following WNV NY99 infection. Protein levels of IL-1β, IL-6, IL-12, IL-10, IL-15, TNF-α, GM-CSF, and G-CSF were assessed in the serum by luminex assay. Data represent the mean concentration (pg/mL) ± SEM (*n* = 6–8 mice per group). * *p* < 0.05.

**Figure 5:**
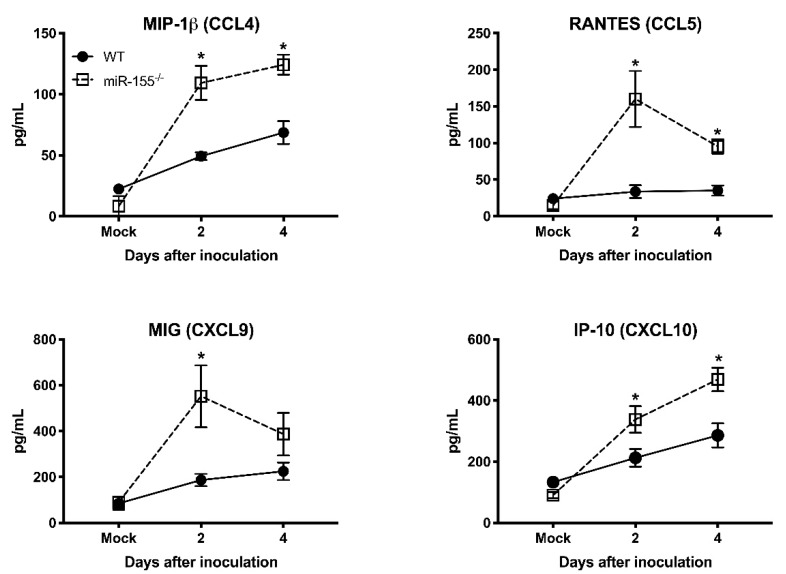
Serum chemokines levels in the WNV NY99-infected miR-155^−/−^ and WT mice. Protein levels of CCL4, CCL5, CXCL9, and CXCL10 were assessed in the serum by luminex assay. Data represent the mean concentration (pg/mL) ± SEM (*n* = 6–8 mice per group). * *p* < 0.05.

It has been shown that pro-inflammatory mediators induced by WNV infection protect mice from lethal WNV disease [27]. To assess the effect of miR-155 deficiency on anti-viral inflammatory response during WNV infection, we measured the systemic protein levels of key pro-inflammatory cytokines and chemokines in miR-155^−/−^ mice infected with WNV NY99. We measured cytokines and chemokines in the same samples used for viremia. The WT mice exhibited high levels of cytokines during WNV infection. However, the absence of miR-155 caused a significant reduction in the cytokine levels at days 2 and 4 after infection (Figure 4). miR-155^−/−^ mice exhibited significantly lower protein levels of interleukin (IL)-1β, IL-6, IL-12, IL-15, and GM-CSF compared to WT mice. However, the protein levels of IL-10, TNF-α, and G-CSF did not differ between both the groups. These data indicate that the knockout of miR-155 in WNV-infected mice resulted in a marked reduction of pro-inflammatory cytokines. Interestingly, protein levels of chemokines involved in immune cell migration were significantly increased in WNV-infected miR-155^−/−^ mice. As depicted in Figure 5, protein levels of CCL4, CCL5, CXCL9, and CXCL10 were significantly higher in miR-155^−/−^ mice compared to WT mice. These data indicate a novel role for miR-155 in regulating the expression of chemokines involved in immune cell migration during WNV infection.

### 3.6. miR-155 Controls WNV Replication in Primary Mouse Cells

**Figure 6:**
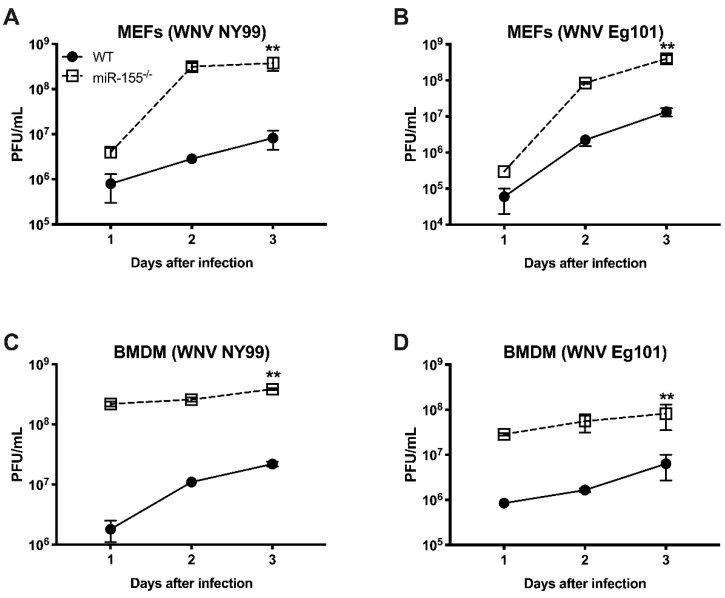
Virus titers in WNV-infected mouse embryonic fibroblasts (MEFs) and bone marrow-derived macrophages (BMDMs) isolated from miR-155^−/−^ and WT mice. (**A**–**D**) MEFs and BMDMs were infected as described in the methods and viral titers in the culture supernatants were assessed by plaque formation assay. The results expressed as PFU/mL ± SEM from three independent experiments conducted in duplicate. ** *p* < 0.001.

In order to further delineate the role of miR-155 in WNV infection, we infected MEFs and BMDMs isolated from WT and miR-155^−/−^ mice with WNV NY99 or WNV Eg101 and assayed virus titers in cell culture supernatants at days 1, 2, and 3 after infection by plaque assay. MEFs from miR-155^−/−^ mice produced significantly higher virus titers compared to those from WT mice after infection with both WNV NY99 and WNV Eg101. At days 2 and 3 after infection, the differences in virus titers between WT and miR-155^−/−^ MEFs were approximately 2 log_10_ for WNV NY99 and 1 log_10_ for WNV Eg101 as represented in Figure 6A,B. Similarly, virus titers were significantly higher in BMDMs lacking miR-155 compared to those from WT mice after infection with either strain of WNV (Figure 6C,D). It is interesting to note that the difference in WNV titers between WT and miR-155^−/−^ cells was much higher in BMDMs (2–3 logs) compared to MEFs at an early time point (24 h) after infection. 

### 3.7. miR-155 Inhibits WNV Replication in Human Neuroblastoma Cells

**Figure 7:**
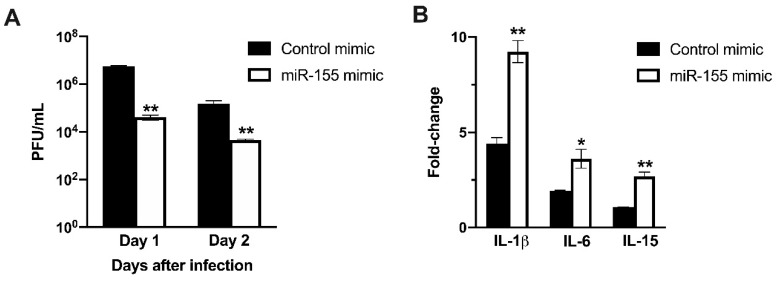
miR-155 mimic inhibits WNV replication in human neuroblastoma cells. (**A**) SK-N-SH cells were transfected with miR-155 mimic or control mimic. Cells were infected with WNV NY99 at a MOI of 1. Viral titers in the cell culture supernatants were assessed by plaque assay and expressed as PFU/ml ± SEM. (**B**) mRNA levels of IL-1β, IL-6, and IL-15 genes were determined using qRT-PCR at 24 h after infection, and the fold change in infected cells compared to corresponding controls was calculated after normalizing to the GAPDH gene. Data represents the mean ± SEM, representing two independent experiments. * *p* < 0.05, ** *p* < 0.001.

To further characterize the role of miR-155 in limiting virus replication and inducing anti-viral immune response, we transfected human neuroblastoma cells, SK-N-SH, with miR-155 mimic or control mimic. We analyzed WNV replication kinetics and host responses in transfected cells. Overexpression of miR-155 in neuronal cells resulted in significantly lower virus replication. As shown in Figure 7A, virus titers in the cells transfected with miR-155 mimic were significantly lower than in cells transfected with control mimic at both 24 and 48 h after WNV NY99 infection. We next determined the mRNA levels of key cytokine genes in transfected cells using qRT-PCR. Transfection of miR-155 induced robust mRNA expression of IL-1β, IL-6, and IL-15 in neuroblastoma cells at 24 h after WNV NY99 infection (Figure 7B). The fold increase of IL-1β, IL-6, and IL-15 were significantly higher in cells transfected with miR-155 mimic than in cells transfected with control mimic. The increase in the expression of IL-6 and IL-1β in miR-155 transfected cells correlated with reduced virus replication. This data supports our in vivo findings where high virus replication in miR-155^−/−^ mice was associated with a lower inflammatory response in the serum. 

## 4. Discussion

Prior studies have reported that miR-155 is a key regulator of host immune and inflammatory responses [39,40,41,42]. We previously reported that WNV infection induces significant upregulation of miR-155 in mice [26]. Herein, we report the critical role of miR-155 in restricting the pathogenesis of WNV infection in mice. miR-155 reduces WNV production in mouse and human cells and impacts anti-WNV immune response. 

In the current study, we observed that WNV NY99-infected miR-155^−/−^ mice displayed higher morbidity and mortality than WT mice. Interestingly, miR-155^−/−^ mice also exhibited 100% mortality after subcutaneous inoculation of a non-pathogenic strain, WNV Eg101. Increased mortality in WNV-infected miR-155^−/−^ mice was associated with a significantly high viral burden in the serum and brains compared to WT mice. In addition, primary mouse cells derived from miR-155^−/−^ mice produced higher WNV titers compared to WT cells. These data indicate impaired clearance of WNV in the periphery and brains of miR-155^−/−^ mice. It has been shown that miR-155 controls HIV infection, whereby the application of miR-155 mimics significantly suppressed HIV replication in activated macrophages [12]. Similar role of miR-155 has been observed in restricting JEV pathogenesis [13,25] and herpes simplex encephalitis [43,44]. In addition, it has been reported that miR-155 regulates viral infections caused by Epstein–Barr, Borna disease, and reticuloendotheliosis viruses [10,11,22].

One interesting finding of our study was the significantly enhanced production of IFN-α in the miR-155^−/−^ mice. miR-155 is multifunctional and modulates various aspects of innate and adaptive immune responses [12,13,19]. Recent reports have demonstrated that miR-155 plays a significant role in regulating type I IFN response [21,45]. The IFN response is crucial for the host defense against WNV infection [46]. In the current study, we observed an enhanced IFN response in miR-155^−/−^ mice compared to WT mice after WNV infection. These data indicate that miR-155 may act as a negative regulator of the type I IFN response during WNV infection. It is also possible that high virus replication in miR-155^−/−^ mice resulted in a higher interferon response in these mice. Collectively, these data suggest that miR-155-mediated restriction of WNV infection is independent of IFN-α. 

Another interesting observation of our study was the significant reduced levels of IL-1β, IL-12, IL-6, IL-15, and GM-CSF in miR-155^−/−^ mice despite the high viral load, suggesting that the deficiency of miR-155 affects the production of anti-viral cytokines. Similarly, our data demonstrated that transfection of miR-155 mimic induced robust mRNA expression of IL-1β, IL-6, and IL-15 in human neuroblastoma cells. It is known that restricted inflammatory response is essential to eliminate pathogens and induce an effective adaptive immune response [46]. IL-1β is a key cytokine that modulates the secretion of other cytokines such as IL-6 [47]. Besides, IL-1β promotes migration of WNV-induced Langerhans cell from the skin to draining lymph nodes in the mice, and also plays an important role in promoting immune cell trafficking into the brain [48,49]. It is known that IL-6 governs antibody production and activation of T cells [50]. Therefore, a possible explanation for the high viral load in the periphery and brains of miR-155^−/−^ mice could be the reduced anti-viral cytokines production in these mice following WNV infection. Several studies have previously reported that miR-155 modulates immune response by promoting cytokine production [51,52].

In contrast to cytokine levels, protein levels of chemokines such as CCL4, CCL5, CXCL9, and CXCL10 were significantly high in WNV-infected miR-155^−/−^ mice than WT mice. It is known that CXCL10, CCL4, and CCL5 promote entry of immune cells including CD4 T cells, CD8 T cells, NK cells, and macrophages into the brain [53]. The high levels of these chemokines in miR-155^−/−^ mice during WNV infection may facilitate increased trafficking of immune cells into the brain [54,55]. Although infiltration of leukocytes in the brain is critical for clearance of WNV, it can also contribute to immunopathology [56] Thus, it is possible that increased chemokine response and leukocyte recruitment in the brains of miR-155^−/−^ mice may contribute to severe disease observed in these mice. The data collectively show that miR-155 possesses an essential function in regulating inflammatory host response during WNV infection.

## 5. Conclusions

In conclusion, our data for the first time revealed the critical role of miR-155 in restricting WNV pathogenesis in mice. miR-155 impacts WNV pathogenesis and resistance and regulates anti-viral cytokine and chemokine responses. There is need for further mechanistic studies to understand how miR-155 restricts WNV infection.

## Figures and Tables

**Table 1:** Primer sequences used for qRT-PCR.

Gene (Accession No.)	Primer Sequence (5′-3′)
**IL-1β (NM_000576)**	
Forward	AGCACCTTCTTTCCCTTCATC
Reverse	GGACCAGACATCACCAAGC
**IL-6 (NM_000600)**	
Forward	CCAGGAGCCCAGCTATGAAC
Reverse	CCCAGGGAGAAGGCAACTG
**IL-15 (NM_172175)**	
Forward	CGAAACCACATTTGAGAA
Reverse	TGAAGGCATTAGTAGAGTAA
